# Illicit drug use and male barroom aggression among members of the Australian construction industry: Associations with personality and masculinity factors

**DOI:** 10.1111/dar.13498

**Published:** 2022-06-14

**Authors:** Steven Litherland, Peter G. Miller, Shannon Hyder

**Affiliations:** ^1^ School of Psychology Deakin University Geelong Australia; ^2^ Centre for Addiction and Mental Health University of Toronto London Canada; ^3^ National Drug Research Institute Curtin University Perth Australia; ^4^ Menzies School of Health Research Charles Darwin University Darwin Australia

**Keywords:** construction workers, illicit drugs, male barroom aggression, masculinity, personality

## Abstract

**Introduction:**

Illicit drug use has been found to increase the risks of male barroom aggression (MBA). Personality traits such as dispositional aggressiveness have been associated with illicit substance use and aggressive behaviour, along with social normative masculinity factors. The present study assessed the relationships between illicit drug use, key personality (trait aggression, impulsivity, narcissism) and masculinity (conformity to masculine norms, male honour) variables with physical MBA perpetration and victimisation among male Australian construction workers.

**Methods:**

A purposive, high‐risk sample of male construction workers aged 18–69 years (*n* = 476, *M*_age_ = 25.90 years, SD_age_ = 9.44) completed interviews at their place of work or training.

**Results:**

Participants reported high rates of both physical MBA perpetration (21%; *n* = 100) and victimisation (31.1%; *n* = 148) as well as any illicit drug use (33.61%; *n* = 160). Logistic regressions revealed the use of amphetamine‐type stimulants (methamphetamine, ecstasy) was associated with violence perpetration, even after accounting for high‐intensity drinking (HID) which was the strongest predictor of MBA involvement. Trait variables (Trait Physical aggressiveness, narcissism) and the masculine norm CMNI Violence were also risk factors for MBA perpetration while CMNI Playboy was protective against MBA.

**Discussion and Conclusions:**

The use of amphetamine‐type stimulants is a risk‐factor for MBA perpetration, as are key personality traits such as aggressiveness and narcissism. Most aspects of masculinity, including male honour, were either unrelated to or protective against involvement in physical violence in bars, clubs or pubs.

## 1 INTRODUCTION

Male barroom aggression (MBA) remains a serious public health and policing issue in Australia [1] with licensed venues regarded as ‘hot spots’ for involvement in physical aggression [2]. While much of the related literature has focused on alcohol as a contributor to violence in or around licensed venues [3, 4], less is known about the influence of illicit drugs, although some research in an Australian context indicates their use can significantly increase the risks of aggression involvement in barroom settings [2].

There is an established link between illicit drug use and interpersonal violence [5], although its influence is typically superseded by alcohol [6]. Alcohol is argued to be more likely to be causally related to aggression [7, 8], with heavy alcohol consumption often a strong contributor to involvement in subsequent violence [3, 4, 9]. The social‐situational influence on drug‐related violence may be especially acute in relation to male barroom aggression given illicit substances are prevalent in the night‐time economy [10]. Illicit drug use on the current night out in or around licensed venues has been shown to increase the likelihood of involvement in aggression [11], with use of such substances argued to contribute more to impulsive than predatory violence perpetration [12], while also being both a pre‐cursor to and a consequence of violent victimisation [13]. The use of multiple substances (polydrug use) has also been shown to be problematic, with some research indicating that it may contribute to physical aggression involvement in bars, clubs or pubs during the previous 3 months [11]. Cocaine's influence on subsequent aggression against strangers is equivocal [14], although there is some evidence of an association with night‐life violence generally [10] and past month victimisation specifically [13]. Findings with respect to cannabis and aggression are also mixed, with some studies suggesting a positive association and others no relationship or even a reduced risk of subsequent violence [11, 15]. Amphetamines have been associated with both physical aggression perpetration toward non‐partners and physical violence victimisation [16]. Specifically, methamphetamine is believed to increase the risks of both defensive and or pre‐emptive aggression [17], however, any association is not consistent with amphetamine‐related violence thought to result from associations between the drug user, the substance and the situation [18], suggesting other personal and contextual factors may be important in who may respond to the use of amphetamines with aggression, including in or around licensed venues.

### 1.1 Trait variables, illicit drugs and aggression

Those most likely to behave aggressively while using illicit drugs are those prone to aggressiveness when not under the influence [6], with trait aggression being a dispositional style marked by a pattern of aggressive behaviour on repeated occasions and across various contexts [19]. Trait aggression is argued to influence and serve as a conduit between drug use and aggression [5, 7, 20]. Both aggression and substance use have been linked with impulsivity; a deficiency in the ability to think ahead and plan carefully, a tendency to engage in rash actions, become easily bored and seek out stimulation or excitement without meaningful consideration of the consequences [21]. Innate aggressiveness and impulsivity have been proposed as core components to certain personality disorders which are often comorbid with substance abuse problems [19]. Narcissism, which reflects highly favourable, even grandiose view of the self and an exaggerated sense of entitlement [22, 23], may also exert an influence on MBA. It is associated with general aggression perpetration [23], particularly in response to ‘wounded pride’ [22], which may be especially salient in licensed venues when under the influence of intoxicants, including illicit drugs.

### 1.2 Masculinity, illicit drugs and aggression

A theoretically important socially constructed factor thought to influence male aggression is masculinity which represents the endorsement of culturally defined beliefs and standards (norms) that are supposedly important to men [24]. Masculine norms are typically operationalised as representing hegemonic or hyper‐masculine ideologies incorporating physical toughness and an endorsement of violence, competitiveness, emotional control, risk‐taking, heterosexuality and anti‐homosexuality [25, 26]. The barroom context is one where conformity to traditional male ideologies can be especially important [27, 28]. It has been argued that certain sub‐cultures also promote both substance use and violence as indicative of ‘masculinity’ [29]. Qualitative evidence has linked masculinity and illicit drug use to violence [30, 31] and quantitatively to sexual aggression victimisation [32], however broader associations with barroom aggression are lacking.

### 1.3 Theoretical framework

The selection of variables is informed by the I^3^ (I‐cubed) theory of aggression [33], which is derived from the General Aggression Model [34, 35]. The I^3^ theory proposes that instigating triggers such as provocation (verbal and or physical) and social rejection can be an influence on or be influenced by impelling variables (e.g. trait aggressiveness, impulsivity and narcissism) as well as (dis)inhibitors in the form of intoxication [33]. These factors determine one's ‘readiness’ to aggress in certain settings such as bars, clubs or pubs. While some research has offered partial support for this theory of aggressive behaviour [36], to date, it has been more theoretical than empirical [33, 37]

### 1.4 Research aims

The selection of variables is consistent with two important components of the I^3^ theory of aggression and aligns with the aims of the present study. That is, to examine the role of potentially important (dis)inhibitors in illicit drug use (amphetamine‐type stimulants [ATS], cocaine and cannabis) as well as impelling factors including key trait variables and masculinity‐related attitudes and beliefs in the prediction of MBA perpetration and victimisation, while also accounting for HID [38] among a high‐risk sample of male Australian construction workers.

It is hypothesized that HID will be the most significant contributor to both physical MBA perpetration and victimisation [H1]. Furthermore, use of illicit drugs (ATS, cocaine or cannabis) [H2], polydrug use [H3] and the use of ATS independently [H4] will be significantly associated with MBA perpetration while trait variables will show stronger associations with reports of barroom violence than masculinity factors [H5].

## 2 METHOD

### 2.1 Participants

The Australian construction industry is male dominated, with men accounting for close to 90% of those employed in the sector [39]. Australian [40] and international [41] research suggests illicit drug use is widespread in the sector and thus, its members likely represent an important sample when seeking to understand if such substances are associated with physical violence perpetration and victimisation in or around licensed venues, particularly given a related sample of Australian tradesmen, many of whom were employed in construction trades, reported high rates of MBA comparative to other populations [4] with blue‐collar workers, including those involved in the building and construction industry, more than twice as likely to be involved in barroom aggression than male professionals [42].

Participants were a purposive sample of 476 male members of the Australian construction industry working or training in Melbourne and greater Geelong, Victoria, Australia, aged 18–69 years (M = 25.90, SD = 9.44). Ninety‐five percent of the people approached agreed to do an interview and all interviews were completed. Sixteen different occupational groups were represented, including plumbers (*n* = 101, 21.2%), bricklayers (*n* = 100, 21%), carpenters (*n* = 92, 19.3%), painters (*n* = 29, 6.1%), electricians (*n* = 22, 4.6%), cabinet makers (*n* = 19, 4%), labourers (*n* = 19, 4%), concreters (*n* = 11, 2.3%) and metal fabricators (*n* = 10, 2.1%).

### 2.2 Procedure

Ethics approval was obtained from the Deakin University Human Ethics Advisory Group‐Health (DUHREC_2013–104). The cross‐sectional design employed face‐to‐face interviews delivered by trained interviewers (comprised of two males and four females). Recruitment occurred at the participants' place of employment (i.e. construction sites) or training (i.e. at TAFE institutions) with all those interviewed having had experience on construction sites. After receiving approval to enter the respective locations by site supervisors or teaching/managerial staff, researchers then approached potential participants by asking them if they would volunteer to participate in the study by undertaking a survey. Those over the age of 18 years who volunteered were then interviewed with data collection occurring between June and November 2016. After providing verbal and written consent, participants were interviewed by a researcher, who recorded all responses on print‐outs of the surveys or electronically using TAP Form's software. Interviews lasted approximately 20 minutes. No reimbursement was given to respondents.

### 2.3 Measures

The 59‐item survey was designed to minimise interview time and limit inconvenience among participants at their place of work or training.

#### 2.3.1 Physical MBA perpetration and victimisation

Participants reported the number of times they had perpetrated physical aggression (You grabbed, pushed or shoved, hit or kicked someone else or did something else physically aggressive) and been victims of physical violence at a bar, club or pub during the previous 12 months, with items drawn from Wells *et al*. [43, 44]. Responses were highly positively skewed and thus converted into dichotomous variables (0 = no, 1 = yes).

#### 2.3.2 Illicit drug use

Participants reported the number of times they had used the following illicit substances: (i) amphetamine‐type stimulants (speed, methamphetamine, ecstasy); (ii) cocaine; and/or (iii) cannabis, in the past month, with these illicit substances being the most prevalent drugs used in the Australian night‐time economy [11]. Responses were highly positively skewed and thus converted into dichotomous variables (0 = no, 1 = yes) for the use of ATS, cocaine and cannabis independently, as well as for any illicit and polydrug use.

#### 2.3.3 High‐intensity drinking

Participants reported the number of times they had consumed eight or more standard alcoholic drinks on a single occasion during the previous month. Responses were dichotomised to form a variable reflecting at least one session of HID (8 or more standard drinks, 80 g of alcohol) in that time (0 = no, 1 = yes).

#### 2.3.4 Trait aggression

The short Aggression Questionnaire (AQ 12) [45] comprises four‐factors; Trait Physical (‘There are people who pushed me so far we came to blows’), Trait Verbal (‘I can't help getting into arguments when people disagree with me’), Trait Anger (‘Sometimes I fly off the handle for no good reason’) and Trait Hostility (‘At times I feel I have gotten a raw deal out of life’). Participants rated the extent to which each item described them, on a Likert‐type scale (1 = extremely uncharacteristic of me, 3 = neutral, 5 = extremely characteristic of me), with scores on each of the three items per subscale averaged.

#### 2.3.5 Narcissism

The Single Item Narcissism Scale [46] asked participants: ‘To what extent do you agree with this statement: I am a narcissist’ (Note: participants were instructed that the word ‘narcissist’ means egotistical, self‐focused and vain) with responses on a 7‐point Likert‐type scale (1 = very untrue, 4 = neither true or untrue, 7 = very true). The Single Item Narcissism Scale correlates positively with several other narcissism measures, is related to both grandiose and vulnerable aspects of the construct, demonstrates convergent and criterion validity with high test–retest reliability and is deemed suitable when the length of a survey is an issue for administrators [46].

#### 2.3.6 Impulsivity

Three items used previously among construction workers in the USA [47] asked participants how well each of the statements described them (e.g. ‘I often act on the spur‐of‐the‐moment’) on a 4‐point Likert‐type scale (1 = not at all, 2 = a little, 3 = some, 4 = quite a lot).

#### 2.3.7 Male honour

Two items derived from Wells *et al*.'s [44] Belief and Attitudes toward Male Alcohol‐Related Aggression (BAMARA) Inventory asked respondents on a 5‐point Likert‐type scale (1 = strongly disagree, 3 = neither, 5 = strongly agree) the extent to which they (dis)agree with the statements: ‘Guys are cowards if they back down from a fight at a bar’ and ‘I'd be ashamed of myself if I didn't stand up to a guy who was threatening to fight me at a bar’. Elements of the BAMARA Inventory have been used reliably in previous studies [3, 48].

#### 2.3.8 Conformity to masculine norms

Eighteen items across six subscales most relevant to MBA were drawn from the Conformity to Masculine Norms Inventory (CMNI)‐46 [49]; Violence (‘Sometimes violent action is necessary’), Winning (‘It is important for me to win’), Emotional Control (‘I tend to keep my feelings to myself’), Risk‐taking (‘I enjoy taking risks’), Playboy (‘I would feel good if I had many sexual partners’) and Heterosexual Self‐presentation (‘It would be awful if people thought I was gay’). The CMNI‐46 is widely used [50, 51] and its subscales have good construct validity and internal reliability [49]. Participants rated the extent to which each item described their own actions, feelings and beliefs on a 4‐point Likert‐type (1 = strongly disagree, 4 = strongly agree) with scores on the three items per‐subscale averaged.

### 2.4 Analyses

Binary logistic regressions examined predictors of any physical MBA perpetration and victimisation. Each of the logistic regressions involved a 3‐step hierarchical modelling process with model 1 featuring drug use types, model 2 including trait and masculinity‐related variables, and model 3 introducing HID. This approach was chosen to gauge preliminary effects of illicit drug use as well as trait and masculinity variables in MBA perpetration and victimisation prior to the inclusion of HID which was likely to exert the most significant influence on involvement in barroom violence.

## 3 RESULTS

Bivariate correlations and internal consistency for all multi‐item measures are presented in Table 1. Overall, the present sample confirmed their at‐risk status with 100 (21%) participants reporting perpetrating physical MBA during the previous 12 months while 148 (31.1%) cited physical victimisation in bars, clubs or pubs. Reports of illicit drug use were high with 160 (33.61%) using ATS, cocaine or cannabis during the previous month with 81 (17.02%) citing use of ATS while 27 (5.67%) and 119 (25%) reported cocaine and cannabis use respectively. Further, 332 (69.75%) participants had engaged in at least one episode of high‐intensity drinking during the previous month.

**TABLE 1:** Descriptive statistics and Pearson correlation coefficient for predictors of physical MBA perpetration and victimisation

Measure	1.	2.	3.	4.	5.	6.	7.	8.	9.	10.	11.	12.	13.	14.	15.	16.	17.	18.	19.	20.	21.
1. MBA Perp (Phys)	1	0.59**	−0.20**	−0.18**	−0.23**	−0.05	−0.12**	0.22**	0.32**	0.22**	0.24**	0.04	0.18**	0.20**	0.12*	0.30**	0.01*	0.19**	−0.00	−0.05	0.20**
2. MBA Vic (Phys)		1	−0.15**	−0.15**	−0.17**	−0.01	−0.11*	0.25**	0.25**	0.23**	0.22**	0.05	0.15**	0.21**	0.13**	0.18**	0.08	0.17**	0.00	−0.08	0.16**
3. ‘Any’ drug use			1	0.56**	0.63**	0.35**	0.81**	−0.28**	−0.23**	−0.18**	−0.17**	−0.11*	−0.07	−0.16**	0.00	−0.22**	−0.02	−0.14**	−0.27**	0.07	−0.14**
4. ‘Polydrug’				1	0.77**	0.48**	0.53**	−0.20**	−0.17**	−0.11*	−0.10*	−0.01	−0.01	−0.16**	−0.06	−0.13**	0.00	−0.14**	−0.14**	0.03	−0.18**
5. ‘ATS’					1	0.35**	0.36**	−0.25**	−0.20**	−0.11*	−0.15**	−0.02	−0.00	−0.15**	0.03	−0.18**	−0.04	−0.15**	−0.16**	0.04	−0.14**
6. Cocaine						1	0.22**	−0.14**	−0.03	−0.06	0.00	−0.01	−0.04	−0.12*	−0.09	−0.01	−0.00	−0.05	−0.09	0.03	−0.03
7. Cannabis							1	−0.18**	−0.23**	−0.16**	−0.14**	−0.13**	−0.04	−0.15**	0.04	−0.17**	0.07	0.09	−0.23**	0.03	−0.20**
8. HID								1	0.14**	0.07	0.11*	0.05	0.04	0.13**	−0.02	0.11*	0.04	0.15**	0.18**	−0.07	0.11
9. Trait Physical									1	0.42**	0.51**	0.32**	0.19**	0.44**	0.12*	0.43**	0.08	0.35**	0.17**	0.12**	0.36**
10. Trait Verbal										1	0.52**	0.30**	0.29**	0.41**	0.27**	0.19**	0.04	0.21**	0.11*	0.09	0.26**
Trait Anger											1	0.42**	0.20**	0.34**	0.11*	0.20**	0.07	0.16**	0.13*	0.09	0.25**
12. Trait Hostility												1	0.15**	0.23**	0.05	0.07	0.05	0.00	0.02	0.16**	0.21**
13.Narcissism													1	0.15**	0.05	0.07	0.05	0.07	0.04	0.02	0.10*
14. Impulsivity														1	0.19**	0.19**	0.14**	0.28**	0.17**	0.05	0.21**
15. CMNI Winning															1	0.17**	0.12*	0.22**	0.12**	0.08	0.13**
16. CMNI Violence																1	0.08	0.29**	0.21**	0.06	0.26**
17. CMNI EC																	1	0.03	0.07	0.06	0.17**
18. CMNI Risk‐taking																		1	0.19**	0.08	0.26**
19. CMNI layboy																			1	0.07	0.15**
20. CMNI HSP																				1	0.21**
21. Male honour																					1
Mean									2.78	2.83	2.70	2.55	2.62	2.14	2.59	2.51	2.51	2.82	2.33	2.28	2.28
SD									0.87	0.82	0.83	0.75	1.43	0.85	0.47	0.51	0.57	0.48	0.52	0.52	0.82
Range									1–5	1–5	1–5	1–5	1–7	1–4	1–4	1–4	1–4	1–4	1–4	1–4	1–5
SR									0.71	0.72	0.73	0.72		0.87	0.61	0.69	0.86	0.81	0.71	0.71	0.60

*Notes*: *P < 0.05; **P < 0.01, two‐tailed. *N* = 476. Physical MBA perpetration (yes = 100, no = 376). Physical MBA victimisation (yes = 148, no = 328). HID (yes = 332, no = 144). ‘Any’ (amphetamine‐type stimulants, cocaine or cannabis) illicit drug use (yes = 160, no = 316). ‘Polydrug’ use (yes = 66, no = 410). Use of amphetamine‐type stimulants (yes = 81, no = 395), cocaine (yes = 27, no = 449) and cannabis (yes = 119, no = 357).

CMNI, Conformity to Masculine Norms Inventory; EC, Emotional Control; HID, high‐intensity drinking; HSP, Heterosexual Self‐presentation; MBA, male barroom aggression; SR, scale reliabilities.

### 3.1 Binary logistic regression models (physical MBA perpetration)

Table 2 presents the models incorporating amphetamine‐type stimulants, cocaine and cannabis independently (step 1), trait and masculinity factors (step 2) as well as HID (step 3) as risk factors for MBA perpetration. Models (1) χ^2^(3) = 23.91, *P* < 0.001, (2) χ^2^(16) = 110.11, *P* < 0.001, and (3) χ^2^(17) = 124.38, *P* < 0.001, were all significant and the Hosmer and Lemeshow Tests were non‐significant, indicating that these variables differentiated perpetrators of barroom violence from non‐perpetrators with classification accuracy (79%, 83% and 83.8%, respectively) and the variance accounted for in the data (Cox & Snell *R* ^2^ = 0.05, 0.21 and 0.23, respectively) improving at every step. The use of ATS was significantly predictive of physical MBA perpetration in each of the models, including in the final model (odds ratio [OR] 2.34, *P* = 0.017) containing all potential explanatory variables with trait physical aggressiveness (OR 1.66, *P* = 0.018), narcissism (OR 1.28, *P =* 0.009), CMNI Violence (OR 3.51, *P* = 0.000) and HID (OR 3.87, *P* = 0.001) also significant risk factors, while CMNI Playboy (OR 0.42, *P* = 0.002) significantly reduced the odds of such violence.

**TABLE 2:** Binary logistic regression for use of ATS, cocaine and cannabis, trait and masculinity variables and HID as predictors of physical male barroom aggression perpetration

	Model 1					Model 2					Model 3				
Variables	β (SE)	Wald χ^2^	Sig.	Exp(B)	95% CI	β (SE)	Wald χ^2^	Sig.	Exp(B)	95% CI	β (SE)	Wald χ^2^	Sig.	Exp(B)	95% CI
ATS	**1.25 (0.30)**	**17.74**	**0.000**	**3.51**	**1.96–6.29**	**1.05 (0.35)**	**8.94**	**0.003**	**2.85**	**1.43–5.65**	**0.85 (0.36)**	**5.71**	**0.017**	**2.34**	**1.17–4.70**
Cocaine	−0.34 (0.49)	0.50	0.482	0.71	0.28–1.84	−0.14 (0.57)	0.06	0.812	0.87	0.29–2.65	−0.24 (0.57)	0.18	0.672	0.78	0.26–2.41
Cannabis	0.25 (0.27)	0.83	0.363	1.28	0.75–2.19	−0.08 (0.33)	0.06	0.802	0.92	0.49–1.75	−0.14 (0.33)	0.17	0.677	0.87	0.45–1.67
Trait Physical						**0.45 (0.21)**	**4.57**	**0.033**	**1.57**	**1.04–2.37**	**0.51 (0.22)**	**5.55**	**0.018**	**1.66**	**1.09–2.54**
Trait Verbal						0.07 (0.20)	0.13	0.715	1.08	0.73–1.59	0.06 (0.21)	0.07	0.786	1.06	0.71–1.58
Trait Anger						0.32 (0.20)	2.65	0.103	1.38	0.94–2.03	0.31 (0.20)	2.30	0.129	1.36	0.91–2.03
Trait Hostility						−0.30 (0.20)	2.43	0.119	0.74	0.50–1.08	−0.36 (0.20)	3.19	0.074	0.70	0.47–1.04
Narcissism						**0.24 (0.09)**	**7.06**	**0.008**	**1.28**	**1.07–1.53**	**0.25 (0.10)**	**6.87**	**0.009**	**1.28**	**1.07–1.55**
Impulsivity						0.06 (0.18)	0.39	0.760	1.06	0.74–1.52	0.02 (0.19)	0.01	0.925	1.02	0.70–1.47
CMNI ‘Winning’						0.18 (0.29)	0.39	0.530	1.20	0.68–2.14	0.30 (0.30)	1.01	0.316	1.36	0.75–2.46
CMNI ‘Violence’						**1.27 (0.33)**	**14.69**	**0.000**	**3.55**	**1.86–6.78**	**1.26 (0.34)**	**13.63**	**0.000**	**3.51**	**1.80–6.84**
CMNI ‘EC’						0.14 (0.24)	0.37	0.543	1.16	0.73–1.84	0.16 (0.24)	0.42	0.517	1.17	0.73–1.87
CMNI ‘Risk‐taking’						0.28 (0.34)	0.69	0.407	1.33	0.68–2.58	0.18 (0.35)	0.26	0.614	1.19	0.60–2.37
CMNI ‘Playboy’						**−0.71 (0.27)**	**6.98**	**0.008**	**0.49**	**0.29–0.83**	**−0.87 (0.28)**	**9.37**	**0.002**	**0.42**	**0.24–0.73**
CMNI ‘HSP’						−0.43 (0.26)	2.75	0.098	0.65	0.39–1.08	−0.33 (0.26)	1.48	0.223	0.72	0.43–1.22
Male honour						0.25 (0.18)	1.88	0.170	1.28	0.90–1.82	0.24 (0.19)	1.68	0.195	1.27	0.89–1.83
HID											**1.35 (0.39)**	**12.00**	**0.001**	**3.87**	**1.80–8.33**

*Note*: *N* = 476. Model (1) χ^2^(3) = 23.91, *P* < 0.001, classification = 79%, Cox & Snell *R* ^2^ = 0.05. Model (2) χ^2^(16) = 110.11, classification = 83%, Cox & Snell *R* ^2^ = 0.21. Model (3) χ^2^(17) = 124.38, *P* < 0.001, classification = 83.8%, Cox & Snell R^2^ = 0.23. *n* = 81 (17.02%) participants reported ‘ATS’ use, n = 27 (5.67%) participants reported ‘cocaine’ use and *n* = 119 (25%) reported ‘cannabis’ use during the previous month.

ATS, amphetamine‐type stimulants; CI, confidence interval; CMNI, Conformity to Masculine Norms Inventory; EC, Emotional Control; HID, high‐intensity drinking (*n* = 332; 69.75%); HSP, Heterosexual Self‐presentation.

### 3.2 Binary logistic regression models (physical MBA victimisation)

Table 3 presents the models incorporating ATS, cocaine and cannabis independently (step 1), trait and masculinity factors (step 2) as well as HID (step 3) as predictors of physical MBA victimisation. Models (1) χ^2^(3) = 14.99, *P* < 0.01, (2) χ^2^(16) = 73.60, *P* < 0.01 and (3) χ^2^(17) = 94.83, *P* < 0.001, were all significant and the Hosmer and Lemeshow Tests non‐significant, indicating that these variables differentiated victims from non‐victims of barroom violence with classification accuracy (68.9%, 72.3% and 73.1%, respectively) and the variance accounted for in the data (Cox & Snell *R* ^2^ = 0.03, 0.14 and 0.18, respectively) improving at every step. Use of ATS significantly predicted barroom violence victimisation in models 1 (OR 2.43, *P* = 0.002) and 2 (OR 1.98, *P* = 0.029) but became non‐significant in the full model after accounting for HID (OR 3.54, *P* = 0.000) which was the only significant risk‐factor with CMNI Playboy (OR 0.56, *P* = 0.012) and CMNI Heterosexual Self‐presentation (OR = 0.62, *P* = 0.033) significantly reducing the risks.

**TABLE 3:** Binary logistic regression for use of ATS, cocaine and cannabis, trait and masculinity variables and HID as predictors of physical male barroom aggression victimisation

	Model 1					Model 2					Model 3				
Variables	β (SE)	Wald χ^2^	Sig.	Exp(B)	95% CI	β (SE)	Wald χ^2^	Sig.	Exp(B)	95% CI	β (SE)	Wald χ^2^	Sig.	Exp(B)	95% CI
ATS	**0.89 (0.28)**	**9.91**	**0.002**	**2.43**	**1.40–4.23**	**0.68 (0.31)**	**4.78**	**0.029**	**1.98**	**1.07–3.64**	0.48 (0.32)	2.31	0.129	1.62	0.87–3.01
Cocaine	−0.53 (0.46)	1.31	0.253	0.59	0.24–1.46	−0.58 (0.51)	1.32	0.251	0.56	0.21–1.51	−0.70 (0.51)	1.91	0.167	0.50	0.18–1.34
Cannabis	0.29 (0.24)	1.38	0.240	1.33	0.83–2.14	0.13 (0.28)	0.21	0.694	1.14	0.66–1.96	0.08 (0.29)	0.08	0.776	1.09	0.62–1.89
Trait Physical						0.20 (0.17)	1.40	0.237	1.22	0.88–1.71	0.24 (0.18)	1.85	0.174	1.27	0.90–1.79
Trait Verbal						0.19 (0.17)	1.32	0.250	1.21	0.87–1.68	0.20 (0.17)	1.40	0.237	1.23	0.88–1.71
Trait Anger						0.24 (0.17)	2.10	0.147	1.27	0.92–1.77	0.23 (0.17)	1.75	0.185	1.26	0.90–1.77
Trait Hostility						−0.22 (0.16)	1.81	0.179	0.80	0.58–1.11	−0.26 (0.17)	2.34	0.126	0.77	0.56–1.08
Narcissism						0.15 (0.08)	3.71	0.054	1.16	1.00–1.35	0.16 (0.08)	3.83	0.050	1.17	1.00–1.37
Impulsivity						0.21 (0.15)	1.98	0.159	1.23	0.92–1.65	0.18 (0.15)	1.42	0.234	1.20	0.89–1.62
CMNI ‘Winning’						0.34 (0.25)	1.92	0.166	1.40	0.87–2.27	0.46 (0.26)	3.25	0.072	1.58	0.96–2.61
CMNI ‘Violence’						0.33 (0.25)	1.73	0.188	1.38	0.85–2.25	0.29 (0.26)	1.27	0.260	1.33	0.81–2.20
CMNI ‘EC’						0.14 (0.20)	0.49	0.483	1.15	0.78–1.68	0.13 (0.20)	0.43	0.513	1.14	0.77–1.69
CMNI ‘Risk‐taking’						0.27 (0.27)	1.06	0.304	1.31	0.78–2.21	0.17 (0.27)	0.37	0.544	1.18	0.69–2.01
CMNI ‘Playboy’						**−0.46 (0.22)**	**4.14**	**0.042**	**0.63**	**0.41–0.98**	**−0.58 (0.23)**	**6.26**	**0.012**	**0.56**	**0.36–0.88**
CMNI ‘HSP’						**−0.54 (0.22)**	**6.03**	**0.014**	**0.59**	**0.38–0.90**	**−0.48 (0.23)**	**4.53**	**0.033**	**0.62**	**0.40–0.96**
Male honour						0.18 (0.15)	1.38	0.240	1.19	0.89–1.60	0.16 (0.15)	1.09	0.297	1.18	0.87–1.59
HID											**1.27 (0.29)**	**18.65**	**0.000**	**3.54**	**2.00–6.29**

*N* = 476. Model (1) χ^2^(3) = 14.99, *P* < 0.01, classification = 68.9%, Cox & Snell *R* ^2^ = 0.03. Model (2) χ^2^(16) = 73.60, *P* < 0.01, Classification = 72.3%, Cox & Snell R^2^ = 0.14. Model (3) χ^2^(17) = 94.83, *P* < 0.001, classification = 73.1%, Cox & Snell *R* ^2^ = 0.18. *n* = 81 participants reported ‘ATS’ use, *n* = 27 (5.67%) participants reported ‘cocaine’ use and *n* = 119 (25%) participants reported ‘cannabis’ use during the previous month.

CI, confidence interval; CMNI, Conformity to Masculine Norms Inventory; EC, Emotional Control; HID, high intensity drinking (*n* = 332; 69.75%); HSP, Heterosexual Self‐presentation.

Cocaine and cannabis were not significantly associated with either physical MBA perpetration or victimisation. Additional analyses also revealed that, when considered collectively, the use of any of the illicit drugs under examination (ATS, cocaine or cannabis) as well as polydrug use (the use of multiple substances) during the previous month were not significantly predictive of physical MBA perpetration after accounting for HID in the final models and became non‐significant in the prediction of physical MBA victimisation after the introduction of other potential explanatory variables into the respective models (see supplementary material for model results).

## 4 DISCUSSION

The present study examined associations between the use of illicit drugs (ATS, cocaine and or cannabis) and MBA involvement among a high‐risk sample of Australian construction workers, while also examining the relationships with key trait variables and masculinity factors. This sample reported high rates of MBA perpetration, MBA victimisation and illicit drug use (see Table 1). Despite the high levels of drug use, and consistent with prior research [16, 52], HID had the strongest association with both physical MBA perpetration and victimisation.

### 4.1 Illicit drugs and male barroom aggression

#### 4.1.1 Amphetamine‐type stimulants and MBA

Using ATS was significantly associated with physical violence perpetration in bars, clubs or pubs, even after accounting for HID, while also being related to victimisation prior to the inclusion of alcohol use in the final model [53]. ATS use has previously been associated with risk‐taking [5] and other behavioural and emotional changes (such as going from friendly to aggressive) [17, 54] which can be especially problematic in bars, clubs or pubs and may help explain the relationship with the perpetration of barroom violence in the present sample. ATS can also increase wakefulness which can lessen the sedating effects of alcohol and heighten alcohol‐related intoxication, which may help explain their significant influence on physical MBA perpetration even after the inclusion of HID in the present study

### 4.2 Trait variables, illicit drugs and male barroom aggression

Trait physical aggression significantly increased the odds of reporting physical MBA perpetration. This is consistent with results observed in previous research [3, 4, 55], and is expected given trait aggressiveness and engagement in illicit substance use share similar predictors [56]. Furthermore, it has been suggested that an aggressive personality may influence a user's ‘drug of choice’, with such selections facilitating key features of temperament and levels of social deviance [57]. An aggressive disposition and drug use association is also thought to interact with context, especially in settings conducive to displays of violence [17, 58], with the bars, clubs or pubs attended by those in the present sample potentially problematic given some substance users with pre‐existing aggressive tendencies may exhibit a proneness for misperceiving or even misattributing potentially volatile scenarios involving ever‐present threats, mostly from strangers or ‘third‐party instigators’ [29]. In general, these findings support elements of the I^3^ model of aggression [35] and suggest the ongoing importance of considering instigating, impelling and inhibiting factors when trying to understand the dynamics of aggressive incidents [59].

The present study also found that increases in narcissism were associated with a 28% increased likelihood of reporting physical MBA perpetration. It may be a likely consequence of the proneness for grandiosity which can draw attention in or around licensed venues, potentially leading to exchanges with others that can escalate into physical violence. Furthermore, some narcissists are susceptible to hyper‐sensitivity with respect to rejection and or perceived criticism, which may be especially acute in barroom environments, particularly when under the influence of intoxicants such as alcohol and illicit drugs given the likely interaction with strangers who may pose an ‘ego threat’ by challenging a narcissist's inflated self‐views in problematic situations which can often arise in licensed venues with their resultant ‘wounded‐pride’ potentially inducing aggressive responses [22, 23].

### 4.3 Masculinity, illicit drugs and male barroom aggression

Of the masculinity factors, this study found that only CMNI Violence was significantly associated with an increased risk of physical MBA perpetration. This 250% increase in perpetration likelihood may be related to the sample, but given their high levels of violent behaviour compared to other samples (especially university students), for some individuals, both substance use and subsequent violence in such settings may serve as affirmation of aspects of ‘masculinity’ in line with perceived cultural norms [29, 60]. Alternatively, CMNI Violence may merely be a proxy for an aggressive temperament as opposed to an essential feature of the masculine identity, a proposition reinforced by its medium bivariate association with trait physical aggressiveness (*r* = 0.43). Endorsement of the CMNI Playboy items (i.e. ‘I would feel good if I had many sexual partners’) was associated with a significantly reduced risk of both perpetrating and being victims of physical MBA. While somewhat unexpected, it may be that members of the current sample put an emphasis on the pursuit of multiple sexual partners rather than engaging in aggression involving other males in or around licensed venues given confrontation is potentially counter‐productive to their primary motivation. Also, it cannot be ruled out that those endorsing the ‘Playboy’ items may engage in more sexual aggression in bars, clubs or pubs as opposed to physical violence with other men. Furthermore, the researchers involved in data collection were of the view that responses to related items may have been a partial reflection of whether or not participants were single or had a partner at the time of interview with those in a relationship less likely to endorse promiscuity than those who were not. This distinction may have exerted an influence on the present findings given responses may have potentially reflected respondents' relationship status rather than an endorsement of a masculine norm that is pervasive in their own lives.

### 4.4 Limitations

The present study limits generalisability to other populations however this sample is particularly suited to this study's aims given their high levels of illicit drug use and involvement in MBA. The present study was cross‐sectional so cannot determine causality. Longitudinal designs may provide more definitive insights into the relationship between illicit drug use and aggression and could be a focus for future researchers. Furthermore, drug use may be used by some participants to alleviate aggressive tendencies and or impulses rather than to heighten such propensities, masking the nature of any observed associations and preventing attempts to determine both cause and effect. Also, using self‐reports can be open to recall error. Furthermore, having participants report MBA perpetration and victimisation is inherently problematic given individuals can be highly subjective about who was the initial aggressor and those involved in such violence can potentially assume multiple roles during the same incident, from peacemaker to perpetrator [10]. However, the differences observed in the present study with respect to the variables associated with perpetration but not victimisation supports our hypothesized effects. The use of brief measures for some constructs (i.e., male honour and narcissism) can also be problematic with respect to reliability however there is a need for brevity in research using face‐to‐face interviews, particularly when responses are sought from participants at their place of employment and or training. Indeed, given the ever‐present problem of research fatigue which can prove detrimental to completion rates, it is important to avoid making surveys too long, repetitive and with items perceived as irrelevant by respondents, thus, the inclusion of fewer, more direct questions may help explain the very high completion rates in the present study and reinforce the reliability of our findings.

## 5 CONCLUSIONS

Illicit drug use is a risk‐factor for MBA, particularly the use of ATS which was strongly associated with increased odds of reporting perpetrating such violence, even after accounting for HID. Trait physical aggression and narcissism are particularly important risk‐factors for such violence. The only masculinity‐related variable to be significantly associated with an increased risk of involvement in barroom aggression was endorsement of the CMNI Violence subscale which may reflect the presence of aggressive tendencies given moderate correlations with key aspects of trait aggression, particularly a propensity for physical violence. Overall, the current findings reinforce the need for multi‐faceted theories of aggression such as the I^3^ theory [35] to guide the selection of potentially relevant variables given the importance of personal, contextual and inhibiting factors. Developing research designs able to delineate the processes involved in substance‐related aggression and the respective influence exerted by individual variables and their interactions is the next step for related studies.

## Supporting information


**Table S1** Binary logistic regression for ‘any’ illicit drug use, trait and masculinity variables and HID as predictors of physical male barroom aggression perpetration.
**Table S2.** Binary logistic regression for ‘polydrug’ use, trait and masculinity variables and HID as predictors of physical male barroom aggression perpetration.
**Table S3.** Binary logistic regression for ‘any’ illicit drug use, trait and masculinity variables and HID as predictors of physical male barroom aggression victimisation.
**Table S4.** Binary logistic regression for ‘polydrug’ use, trait and masculinity variables and HID as predictors of physical male barroom aggression victimisation.
