# Supplementary material for: Illicit drug use and male barroom aggression among members of the Australian construction industry: Associations with personality and masculinity factors
Source: Drug Alcohol Rev. 2022 Jun 14;41(6):1463–74. doi: 10.1111/dar.13498 (PMC9542170; doi:10.1111/dar.13498)
Supplement: Supplementary file 1 — Table S1 Binary logistic regression for ‘any’ illicit drug use, trait and masculinity variables and HID as predictors of physical male barroom aggression perpetration. Table S2. Binary logistic regression for ‘polydrug’ use, trait and masculinity variables and HID as predictors of physical male barroom aggression perpetration. Table S3. Binary logistic regression for ‘any’ illicit drug use, trait and masculinity variables and HID as predictors of physical male barroom aggression victimisation. Table S4. Binary logistic regression for ‘polydrug’ use, trait and masculinity variables and HID as predictors of physical male barroom aggression victimisation. [file DAR-41-1463-s001.docx]

Table S1. Binary logistic regression for ‘any’ illicit drug use, trait and masculinity variables and HID as predictors of physical male barroom aggression perpetration

|  | **Model 1** | | | | | **Model 2** | | | | | **Model 3** | | | | |
| --- | --- | --- | --- | --- | --- | --- | --- | --- | --- | --- | --- | --- | --- | --- | --- |
| **Variables** | **β (SE)** | **Wald χ^2^** | **Sig.** | **Exp(B)** | **95% CI** | **β (SE)** | **Wald χ^2^** | **Sig.** | **Exp(B)** | **95% CI** | **β (SE)** | **Wald χ^2^** | **Sig.** | **Exp(B)** | **95% CI** |
| ‘Any’ drug use | **0.99 (0.23)** | **18.43** | **0.000** | **2.69** | **1.71-4.22** | **0.65 (0.28)** | **5.51** | **0.019** | **1.92** | **1.11-3.31** | 0.44 (0.29) | 2.34 | 0.126 | 1.55 | 0.88-2.72 |
| Trait Physical |  |  |  |  |  | **0.46 (0.21)** | **4.82** | **0.028** | **1.58** | **1.05-2.37** | **0.52 (0.21)** | **5.85** | **0.016** | **1.68** | **1.10-2.55** |
| Trait Verbal |  |  |  |  |  | 0.02 (0.20) | 0.01 | 0.935 | 1.02 | 0.69-1.50 | 0.00 (0.20) | 0.00 | 0.992 | 1.00 | 0.67-1.49 |
| Trait Anger |  |  |  |  |  | 0.36 (0.20) | 3.33 | 0.068 | 1.43 | 0.97-2.11 | 0.35 (0.20) | 3.06 | 0.080 | 1.43 | 0.96-2.12 |
| Trait Hostility |  |  |  |  |  | -0.35 (0.19) | 3.29 | 0.070 | 0.70 | 0.48-1.03 | -0.39 (0.20) | 3.85 | 0.050 | 0.68 | 0.46-1.00 |
| Narcissism |  |  |  |  |  | **0.23 (0.09)** | **6.39** | **0.012** | **1.26** | **1.05-1.50** | **0.24 (0.09)** | **6.48** | **0.011** | **1.27** | **1.06-1.53** |
| Impulsivity |  |  |  |  |  | 0.06 (0.18) | 0.11 | 0.742 | 1.06 | 0.75-1.51 | 0.01 (0.19) | 0.00 | 0.974 | 1.01 | 0.70-1.45 |
| CMNI winning |  |  |  |  |  | 0.22 (0.29) | 0.60 | 0.440 | 1.25 | 0.71-2.21 | 0.34 (0.30) | 1.28 | 0.258 | 1.41 | 0.78-2.54 |
| CMNI violence |  |  |  |  |  | **1.19 (0.33)** | **13.36** | **0.000** | **3.30** | **1.74-6.26** | **1.21 (0.34)** | **12.88** | **0.000** | **3.36** | **1.73-6.52** |
| CMNI EC |  |  |  |  |  | 0.14 (0.23) | 0.38 | 0.538 | 1.15 | 0.73-1.82 | 0.17 (0.24) | 0.50 | 0.479 | 1.18 | 0.74-1.88 |
| CMNI risk-taking |  |  |  |  |  | 0.37 (0.34) | 1.19 | 0.276 | 1.44 | 0.75-2.80 | 0.26 (0.35) | 0.54 | 0.463 | 1.29 | 0.65-2.55 |
| CMNI playboy |  |  |  |  |  | **-0.73 (0.27)** | **7.49** | **0.006** | **0.48** | **0.29-0.81** | **-0.86 (0.28)** | **9.50** | **0.002** | **0.42** | **0.24-0.73** |
| CMNI HSP |  |  |  |  |  | -0.40 (0.26) | 2.46 | 0.117 | 0.67 | 0.41-1.11 | -0.30 (0.26) | 1.31 | 0.253 | 0.74 | 0.44-1.24 |
| Male honour |  |  |  |  |  | 0.23 (0.18) | 1.75 | 0.186 | 1.26 | 0.90-1.77 | 0.21 (0.18) | 1.38 | 0.240 | 1.24 | 0.87-1.76 |
| HID |  |  |  |  |  |  |  |  |  |  | **1.39 (0.39)** | **12.80** | **0.000** | **4.00** | **1.87-8.56** |

N=476. Model (1) χ^2^(1)=18.38, *P* <0.001, Classification=79%, Cox & Snell R^2^=0.04 . Model (2) χ^2^(14)=105.48, *P* <0.001, Classification=82.8%, Cox & Snell R^2^=0.20. Model (3) χ^2^(15)=120.81, *P* <0.001, Classification=83.8%, Cox & Snell R^2^=0.22. N=160 (33.61%) participants reported ‘any’ illicit drug use over the previous month (amphetamine-type stimulants, cocaine or cannabis). CI, confidence interval; CMNI, Conformity to Masculine Norms Inventory; EC, emotional control; HID, high intensity drinking (N=332; 69.75%); HSP, heterosexual self-presentation.

Table S2. Binary logistic regression for ‘polydrug’ use, trait and masculinity variables and HID as predictors of physical male barroom aggression perpetration

|  | **Model 1** | | | | | **Model 2** | | | | | **Model 3** | | | | |
| --- | --- | --- | --- | --- | --- | --- | --- | --- | --- | --- | --- | --- | --- | --- | --- |
| **Variables** | **β (SE)** | **Wald χ^2^** | **Sig.** | **Exp(B)** | **95% CI** | **β (SE)** | **Wald χ^2^** | **Sig.** | **Exp(B)** | **95% CI** | **β (SE)** | **Wald χ^2^** | **Sig.** | **Exp(B)** | **95% CI** |
| ‘Polydrug’ use | **1.08 (0.28)** | **14.65** | **0.000** | **2.95** | **1.70-5.14** | **0.79 (0.33)** | **5.59** | **0.018** | **2.20** | **1.14-4.23** | 0.58 (0.34) | 2.85 | 0.091 | 1.78 | 0.91-3.48 |
| Trait Physical |  |  |  |  |  | **0.46 (0.21)** | **4.80** | **0.029** | **1.58** | **1.05-2.38** | **0.52 (0.22)** | **5.75** | **0.017** | **1.67** | **1.10-2.55** |
| Trait Verbal |  |  |  |  |  | 0.05 (0.20) | 0.07 | 0.789 | 1.05 | 0.72-1.55 | 0.03 (0.20) | 0.03 | 0.869 | 1.03 | 0.70-1.54 |
| Trait Anger |  |  |  |  |  | 0.37 (0.20) | 3.48 | 0.062 | 1.44 | 0.98-2.12 | 0.35 (0.20) | 3.04 | 0.081 | 1.42 | 0.96-2.11 |
| Trait Hostility |  |  |  |  |  | -0.31 (0.19) | 2.52 | 0.112 | 0.74 | 0.50-1.08 | -0.36 (0.20) | 3.29 | 0.070 | 0.70 | 0.47-1.03 |
| Narcissism |  |  |  |  |  | **0.24 (0.09)** | **6.94** | **0.008** | **1.27** | **1.06-1.52** | **0.24 (0.09)** | **6.72** | **0.010** | **1.28** | **1.06-1.54** |
| Impulsivity |  |  |  |  |  | 0.04 (0.18) | 0.05 | 0.830 | 1.04 | 0.73-1.48 | -0.01 (0.19) | 0.00 | 0.970 | 0.99 | 0.69-1.43 |
| CMNI ‘winning’ |  |  |  |  |  | 0.15 (0.29) | 0.27 | 0.607 | 1.16 | 0.66-2.05 | 0.29 (0.30) | 0.91 | 0.341 | 1.33 | 0.74-2.39 |
| CMNI ‘violence’ |  |  |  |  |  | **1.30 (0.33)** | **15.92** | **0.000** | **3.68** | **1.94-6.99** | **1.28 (0.34)** | **14.54** | **0.000** | **3.61** | **1.87-6.99** |
| CMNI ‘EC’ |  |  |  |  |  | 0.19 (0.23) | 0.62 | 0.430 | 1.20 | 0.76-1.90 | 0.20 (0.24) | 0.68 | 0.409 | 1.22 | 0.76-1.94 |
| CMNI ‘risk-taking’ |  |  |  |  |  | 0.31 (0.34) | 0.86 | 0.354 | 1.36 | 0.71-2.63 | 0.21 (0.35) | 0.36 | 0.547 | 1.23 | 0.62-2.43 |
| CMNI ‘playboy’ |  |  |  |  |  | **-0.67 (0.26)** | **6.43** | **0.011** | **0.51** | **0.31-0.86** | **-0.84 (0.28)** | **9.05** | **0.003** | **0.43** | **0.25-0.75** |
| CMNI ‘HSP’ |  |  |  |  |  | -0.43 (0.26) | 2.83 | 0.092 | 0.65 | 0.39-1.07 | -0.32 (0.26) | 1.46 | 0.227 | 0.73 | 0.43-1.22 |
| Male honour |  |  |  |  |  | 0.20 (0.18) | 1.33 | 0.249 | 1.23 | 0.87-1.73 | 0.19 (0.18) | 1.13 | 0.288 | 1.21 | 0.85-1.73 |
| HID |  |  |  |  |  |  |  |  |  |  | **1.40 (0.39)** | **13.13** | **0.000** | **4.06** | **1.90-8.66** |

N=476. Model (1) χ^2^(1)=13.74, *P* <0.001, Classification=79%, Cox & Snell R^2^=0.03. Model (2) χ^2^(14)=105.43, *P* <0.001, Classification=81.9%, Cox & Snell R^2^=.20. Model (3) χ^2^=121.28, *P* <0.001, Classification=82.6%, Cox & Snell R^2^=0.23. N=66 (13.87%) participants reported ‘polydrug’ use during the previous month. CI, confidence interval; CMNI, Conformity to Masculine Norms Inventory; EC, emotional control; HID, high intensity drinking (N=332; 69.75%); HSP, heterosexual self-presentation.

Table S3. Binary logistic regression for ‘any’ illicit drug use, trait and masculinity variables and HID as predictors of physical male barroom aggression victimization

|  | **Model 1** | | | | | **Model 2** | | | | | **Model 3** | | | | |
| --- | --- | --- | --- | --- | --- | --- | --- | --- | --- | --- | --- | --- | --- | --- | --- |
| **Variables** | **β (SE)** | **Wald χ^2^** | **Sig.** | **Exp(B)** | **95% CI** | **β (SE)** | **Wald χ^2^** | **Sig.** | **Exp(B)** | **95% CI** | **β (SE)** | **Wald χ^2^** | **Sig.** | **Exp(B)** | **95% CI** |
| ‘Any’ drug use | **0.65 (0.21)** | **10.08** | **0.001** | **1.92** | **1.28-2.87** | 0.39 (0.24) | 2.65 | 0.104 | 1.47 | 0.92-2.35 | 0.17 (0.25) | 0.48 | 0.488 | 1.19 | 0.73-1.92 |
| Trait Physical |  |  |  |  |  | 0.22 (0.17) | 1.67 | 0.196 | 1.24 | 0.89-1.73 | 0.25 (0.17) | 2.10 | 0.148 | 1.29 | 0.92-1.81 |
| Trait Verbal |  |  |  |  |  | 0.17 (0.17) | 1.05 | 0.307 | 1.18 | 0.86-1.64 | 0.19 (0.17) | 1.24 | 0.266 | 1.21 | 0.87-1.69 |
| Trait Anger |  |  |  |  |  | 0.28 (0.17) | 2.76 | 0.097 | 1.32 | 0.95-1.83 | 0.26 (0.17) | 2.34 | 0.127 | 1.30 | 0.93-1.82 |
| Trait Hostility |  |  |  |  |  | -0.25 (0.16) | 2.24 | 0.135 | 0.78 | 0.57-1.08 | -0.27 (0.17) | 2.59 | 0.108 | 0.76 | 0.55-1.06 |
| Narcissism |  |  |  |  |  | 0.14 (0.08) | 3.19 | 0.074 | 1.15 | 1.00-1.33 | 0.15 (0.08) | 3.43 | 0.064 | 1.16 | 0.99-1.35 |
| Impulsivity |  |  |  |  |  | 0.20 (0.15) | 1.84 | 0.175 | 1.22 | 0.92-1.63 | 0.16 (0.15) | 1.16 | 0.283 | 1.18 | 0.87-1.58 |
| CMNI winning |  |  |  |  |  | 0.32 (0.24) | 1.76 | 0.185 | 1.38 | 0.86-2.23 | 0.43 (0.25) | 2.87 | 0.090 | 1.54 | 0.94-2.53 |
| CMNI violence |  |  |  |  |  | 0.33 (0.25) | 1.83 | 0.176 | 1.39 | 0.86-2.26 | 0.31 (0.25) | 1.50 | 0.221 | 1.36 | 0.83-2.24 |
| CMNI EC |  |  |  |  |  | 0.13 (0.19) | 0.47 | 0.494 | 1.14 | 0.78-1.67 | 0.13 (0.20) | 0.42 | 0.516 | 1.14 | 0.77-1.68 |
| CMNI risk-taking’ |  |  |  |  |  | 0.29 (0.26) | 1.21 | 0.271 | 1.34 | 0.80-2.25 | 0.18 (0.27) | 0.45 | 0.502 | 1.20 | 0.71-2.04 |
| CMNI playboy |  |  |  |  |  | **-0.47 (0.22)** | **4.34** | **0.037** | **0.63** | **0.41-0.97** | **-0.58 (0.23)** | **6.15** | **0.013** | **0.56** | **0.36-0.89** |
| CMNI HSP |  |  |  |  |  | **-0.53 (0.22)** | **5.95** | **0.015** | **0.59** | **0.38-0.90** | **-0.48 (0.23)** | **4.58** | **0.032** | **0.62** | **0.40-0.96** |
| Male honour |  |  |  |  |  | 0.19 (0.15) | 1.66 | 0.198 | 1.21 | 0.91-1.61 | 0.17 (0.15) | 1.25 | 0.263 | 1.19 | 0.88-1.59 |
| HID |  |  |  |  |  |  |  |  |  |  | **1.27 (0.29)** | **18.85** | **0.000** | **3.56** | **2.01-6.31** |

N=476. Model (1) χ^2^(1)=10.02, *P* <0.01, Classification=68.9%, Cox & Snell R^2^=0.02. Model (2) χ^2^(14)=70.27, *P* <0.001, Classification=73.5%, Cox & Snell R^2^=0.14. Model (3) χ^2^(15)=91.79, *P* <0.001, Classification=74.4%, Cox & Snell R^2^=0.18. N=160 (33.61%) participants reported ‘any’ illicit drug use during the previous month (amphetamine-type stimulants, cocaine or cannabis). CI, confidence interval; CMNI, Conformity to Masculine Norms Inventory; EC, emotional control; HID, high intensity drinking (N=332; 69.75%); HSP, heterosexual self-presentation.

Table S4. Binary logistic regression for ‘polydrug’ use, trait and masculinity variables and HID as predictors of physical male barroom aggression victimisation

|  | **Model 1** | | | | | **Model 2** | | | | | **Model 3** | | | | |
| --- | --- | --- | --- | --- | --- | --- | --- | --- | --- | --- | --- | --- | --- | --- | --- |
| **Variables** | **β (SE)** | **Wald χ^2^** | **Sig.** | **Exp(B)** | **95% CI** | **β (SE)** | **Wald χ^2^** | **Sig.** | **Exp(B)** | **95% CI** | **β (SE)** | **Wald χ^2^** | **Sig.** | **Exp(B)** | **95% CI** |
| ‘Polydrug’ use | **0.87 (0.27)** | **10.40** | **0.001** | **2.39** | **1.41-4.05** | 0.56 (0.30) | 3.53 | 0.060 | 1.76 | 0.98-3.15 | 0.37 (0.31) | 1.42 | 0.233 | 1.44 | 0.79-2.62 |
| Trait Physical |  |  |  |  |  | 0.21 (0.17) | 2.59 | 0.208 | 1.24 | 0.89-1.73 | 0.25 (0.17) | 1.97 | 0.160 | 1.28 | 0.91-1.80 |
| Trait Verbal |  |  |  |  |  | 0.19 (0.17) | 1.27 | 0.259 | 1.21 | 0.87-1.67 | 0.20 (0.17) | 1.37 | 0.242 | 1.22 | 0.87-1.70 |
| Trait Anger |  |  |  |  |  | 0.28 (0.17) | 2.80 | 0.094 | 1.32 | 0.95-1.83 | 0.26 (0.17) | 2.32 | 0.128 | 1.30 | 0.93-1.82 |
| Trait Hostility |  |  |  |  |  | -0.22 (0.16) | 1.75 | 0.186 | 0.81 | 0.59-1.11 | -0.26 (0.17) | 2.30 | 0.129 | 0.78 | 0.56-1.08 |
| Narcissism |  |  |  |  |  | 0.14 (0.08) | 3.42 | 0.065 | 1.15 | 0.99-1.34 | 0.15 (0.08) | 3.53 | 0.060 | 1.16 | 0.99-1.36 |
| Impulsivity |  |  |  |  |  | 0.18 (0.15) | 1.53 | 0.216 | 1.20 | 0.90-1.61 | 0.15 (0.15) | 1.01 | 0.314 | 1.17 | 0.87-1.57 |
| CMNI winning |  |  |  |  |  | 0.29 (0.24) | 1.37 | 0.242 | 1.33 | 0.83-2.14 | 0.41 (0.25) | 2.65 | 0.104 | 1.51 | 0.92-2.47 |
| CMNI violence |  |  |  |  |  | 0.38 (0.25) | 2.35 | 0.125 | 1.46 | 0.90-2.35 | 0.33 (0.25) | 1.70 | 0.193 | 1.39 | 0.85-2.28 |
| CMNI EC |  |  |  |  |  | 0.15 (0.19) | 0.62 | 0.431 | 1.17 | 0.80-1.71 | 0.14 (0.20) | 0.51 | 0.476 | 1.15 | 0.78-1.70 |
| CMNI risk-taking |  |  |  |  |  | 0.28 (0.27) | 1.11 | 0.292 | 1.32 | 0.79-2.22 | 0.17 (0.27) | 0.40 | 0.527 | 1.19 | 0.70-2.02 |
| CMNI playboy |  |  |  |  |  | -0.43 (0.22) | 3.76 | 0.053 | 0.65 | 0.42-1.01 | **-0.57 (0.23)** | **6.18** | **0.013** | **0.57** | **0.36-0.89** |
| CMNI HSP |  |  |  |  |  | **-0.55 (0.22)** | **6.34** | **0.012** | **0.58** | **0.38-0.89** | **-0.49 (0.23)** | **4.68** | **0.030** | **0.62** | **0.40-0.96** |
| Male honour |  |  |  |  |  | 0.17 (0.15) | 1.31 | 0.253 | 1.19 | 0.89-1.58 | 0.16 (0.15) | 1.04 | 0.308 | 1.17 | 0.87-1.57 |
| HID |  |  |  |  |  |  |  |  |  |  | **1.26 (0.29)** | **18.87** | **0.000** | **3.53** | **2.00-6.25** |

N=476. Model (1) χ^2^(1)=10.18, *P* <0.01, Classification=68.9%, Cox & Snell R^2^=0.02. Model (2) χ^2^(14)=71.12, *P* <0.001, Classification=73.5%, Cox & Snell R^2^=.14. Model (3) χ^2^(15)=92.72, *P* <0.001, Classification=73.9%, Cox & Snell R^2^=0.18. N=66 (13.87%) participants reported ‘polydrug’ use during the previous month. CI, confidence interval; CMNI, Conformity to Masculine Norms Inventory; EC, emotional control; HID, heavy intensity drinking (N=332; 69.75%); HSP, heterosexual self-presentation.
